# Adiponectin, leptin and TNF-α serum levels in obese and normal weight Peruvian adults with and without chronic periodontitis

**DOI:** 10.4317/jced.52350

**Published:** 2015-07-01

**Authors:** Gerardo Mendoza-Azpur, Carmen Castro, Lizet Peña, Maria-Eugenia Guerrero, Manuel De La Rosa, Claudio Mendes, Leandro Chambrone

**Affiliations:** 1 Department of Periodontology, School of Dentistry, Cientifica del Sur University, Lima, Peru; 2 Private dental Practice, Lima, Peru; 3 Department of Periodontology, School of Dentistry, University of Monterrey, Monterrey, Mexico; 4 Division of Periodontics, Department of Stomatology, School of Dentistry, University of São Paulo, São Paulo, SP, Brazil; 5 Department of Periodontics, College of Dentistry, The University of Iowa, USA; and Unit of Basic Oral Investigation (UIBO), School of Dentistry, El Bosque University, Colombia

## Abstract

**Background:**

TNF-α, an adipokine involved in systemic inflammation and a member of a group of cytokines that stimulate the acute phase reaction, has been related to the pathogenesis of both periodontitis and obesity. The objective of this study was to assess the serum levels of adiponectin, leptin and TNF-α of periodontally healthy normal weight (NW) patients, NW patients with chronic periodontitis (CP), periodontally healthy obese patients and obese patients with CP.

**Material and Methods:**

Ninety-three patients were enrolled in this cross-sectional study: 30 periodontally healthy NW patients; 18 NW patients with CP; 21 periodontally healthy obese patients; and 24 obese patients with CP. Analyses included clinical and anthropometric outcomes, as well as the assessment of serum levels of adiponectin, leptin and TNF-α by enzyme linked immunosorbent assay (ELISA) or fully automated chemiluminescence immunoassay. One-Way ANOVA, Kruskal-Wallis One-Way on Ranks, Dunn’s Test and multivariable logistic regression (MLR) analyses were conducted to estimate the degree of association between periodontitis and obesity.

**Results:**

Obese patients with CP showed significant more bleeding sites than the other three groups (*p*<0.05). Moreover, patients from the NWCP and OPH showed similar BOP percentages, as well as OPH group showed more bleeding sites than the NWPH group (*p*<0.05). The OPH group showed similar levels of adiponectin and leptin than the OCP group, but significantly higher than the NWPH and NWCP groups(*p*<0.05). MLR analyses showed that obesity was positively associated with the percentage of sites with bleeding on probing, with an odds ratio of 0.93 (95% confidence interval: -0.88, - 0.98; *p*=0.012).

**Conclusions:**

The serum levels of adiponectin, leptin and TNF-α were not influenced by CP. Obese patients showed almost 10% more sites with BoP. In chronic periodontitis patients, obese subjects presented significant more BOP sites than normal weight subjects.

** Key words:**Periodontitis, obesity, inflammation, adiponectin, leptin.

## Introduction

It is well-established that the development and progression of chronic periodontitis (CP) is directly linked to dental biofilm accumulation on dental surfaces (1-3). If left undisturbed, dental plaque biofilms stimulate the inflammation of the periodontal tissue, as well as an activation of a ceaseless host response that may trigger periodontal destruction (1). Despite being focal and confined to the oral cavity, CP generates an inflammatory burden that may interact with other body environments, systemic diseases and conditions, such as adverse pregnancy outcomes (4), atherosclerotic cardiovascular disease (5), chronic kidney disease (6) and diabetes mellitus (7,8).

The progression of CP may also be affected by other conditions such as smoking (9-11) and diabetes mellitus (7). It has been suggested, that this complex and concomitant interplay of different biologic processes seems to associate periodontitis to obesity (12). Obesity, defined by the World Health Organization (WHO) (13) was abnormal or excessive fat accumulation that may impair health’, is evaluated by the body mass index (BMI), i.e. a person’s weight in kilograms divided by the square of his height in meters (kg/m2). The WHO estimates that more than 1.4 billion adults > 20 years of age are overweight, and out of this number, more than of 500 million are obese. Greater than 2.8 million people die every year as a consequence of being overweight and/or obesity. These conditions are also associated to 44% of the diabetes mellitus burden and 23% of the population suffering from ischemic heart disease (13).

Efforts have been made to comprehend the endocrine features of white adipose tissue and its role in the production of two important adipocytokines: adinopectin and leptin (14). The first is an anti-inflammatory protein that modulates glucose metabolism, fatty acids, and immune response, whereas the second is a pro-inflammatory peptide hormone that regulates food intake, energy balance, fat storage and the secretion of other cytokines (14,15). Reduced serum concentrations of adiponectin are associated with obesity, insulin resistance and type 2 diabetes mellitus (14,16). Conversely, obese subject may present elevated serum leptin levels (17).

Systematic review outcomes revealed that obese subjects may present more attachment loss than non-obese subjects, and that patients with periodontitis reported greater body mass indexes (BMI) than periodontally healthy subjects (18,19). These studies also suggested that chronic inflammatory processes occurring in the periodontium of patients with periodontal disease may lead to systemic inflammation and secretion of locally produced pro-inflammatory cytokines/mediators (18,19). TNF-α, an adipokine involved in systemic inflammation and a member of a group of cytokines that stimulate the acute phase reaction, has been related to the pathogenesis of both periodontitis (20) and obesity (21).

Previous studies have reported increased serum levels of leptin in patients with periodontitis (17,22). More recent investigations (23) reported that periodontitis was associated with reduced levels of serum adiponectin, and that obesity and periodontitis may regulate the serum levels of leptin in favor of proinflammation. In addition, these authors found amongst subjects with periodontitis,obese or normal weighing, a significant increase in TNF-α levels in sites with probing depths greater than 5mm.

Thus, the objective of this study was to assess the serum levels of adiponectin, leptin and TNF-α of periodontally healthy (PH) normal weight (NW) subjects, NW subjects with chronic periodontitis (CP), PH obese subjects and obese subjects with CP.

## Material and Methods

-Ethical Consideration and Study Design

This cross-sectional study was approved by the Ethics on Research Committee of the Faculty of Dentistry at the Científica del Sur University, and was conducted in accordance with the Helsinki Declaration of 1975 as revised in the year 2000. The subjects participating in the study were volunteers who received detailed information regarding the proposed research and provided signed informed consent. In addition, the study design was prepared according to The Strengthening the Reporting of Observational Studies in Epidemiology (STROBE) statement (24).

-Study Population and Inclusion Criteria

Ninety three systemically healthy, non-smoking subjects, 25 male and 68 female, 30 to 60 years old (mean age: 42.6 years), participated in the study. These subjects were selected from an initial sample of 600 patients who were referred for dental treatment at the dental clinic of Científica del Sur University and at two private practices between July 2012 and January 2013. The patients were enrolled in the study when they met the following inclusion criteria: 1) age > 30 years; 2) at least 15 teeth, excluding third molars; 3) non-smoking status; and 4) a normal weight or obese type I or II status (13). All consecutive patients who met these inclusion criteria were invited to participate in the study. Patients with a diagnosis of gingivitis or aggressive periodontitis, a known systemic disease such asacquired immunodeficiency syndrome [AIDS] or diabetes mellitus, pregnant/lactating, currently undergoing orthodontic treatment or submitted to periodontal therapy within the previous 12 months, or having undergone antimicrobial, anti-inflammatory and/or immunosuppressive therapies during the previous 6 months were excluded from the study.

-BMI Assessment 

BMI was calculated based on each subject’s weight in kilograms divided by the square of his height in meters (kg/m2). Weight and height measurements were performed by the same trained examiners. Subjects were classified as obese if they presented. Obesity was defined according to the WHO (2012) as type I (i.e. BMI greater than or equal to 30 kg/m2 but less than35 kg/m2) or type II (i.e. BMI greater than or equal to 35 kg/m2 but less than 40 kg/m2). NW was defined as BMI ranging from 20 to 24.9 kg/m2.

-Clinical examination 

Full medical and dental histories were obtained. Data included full mouth probing depth (PD) measured at six sites around the teeth, clinical attachment level (CAL) and presence or absence of bleeding on probing using a UNC-15 periodontal probe recorded by two trained and calibrated periodontists. (kappa intra-class correlation coefficient > 0.82 for PD and CAL). Patients without a history of periodontitis, no sites with PD and CAL > 3 mm concomitantly were included in the periodontally healthy groups. Those exhibiting bleeding on probing in at least four teeth with a PD >4 mm were entered to the chronic periodontitis group. Chronic periodontitis (CP) was defined according to the criteria established by the World Workshop and the American Academy of Periodontology (25).

-Sample Size Calculation and Experimental Groups

Sample size calculation was performed based on the findings of a previous study (23). The primary outcome was considered TNF alpha levels. Considering an expected standard deviation of 2.8 pg/ml, the required number of subjects to detect a relevant mean difference of 2.5 pg/ml between any two of the four groups (alpha = 5%, power = 80%) was estimated at 17 patients for each group.

Following the assessment of clinical and BMI outcomes, subjects were divided into four groups: 1) NWPH (n=30): NW subjects without CP; 2) NWCP (n=18): NW subjects with CP; 3) OPH (n=21): Obese subjects without CP; and 4) OCP (n=24): Obese subjects with CP.

-Serum sampling

Serum samples were obtained as described in a previous investigation (23). Peripheral blood samples were collected in the mor-ning and one week after clinical examination into a proper tube. Subsequently, the serum was separated from blood by centrifugation (10 min at 1,300 rpm) and stored at -80°C. Serum levels of adiponectin and leptin were analyzed by enzyme linked immunosorbent assay (ELISA), as well as TNF-α by fully automated chemiluminescence immunoassay. The ranges of detection for adiponectin, leptin and TNF-α were 2.0 to 20.0 ug/ml (male) and 4.0 to 22.0 ug/ml (female), 1.0 to 10.0 ng/ml (male) and 4.0 to 25.0 ng/ml (female), and 2.0 to 7.8 pg/ml, respectively. In addition, serum sampling was performed at a biomedical laboratory contracted for such specific purpose, and sample tubes were identified only by the name of the subject.

-Statistical Analysis

Descriptive statistics were used to synthesize collected data. The modified-Levene test was applied to test for equality of variance. Differences between the groups for Age, BoP, CAL, adiponectin, leptin, and TNF-α were analysed using One-Way Analysis of Variance (ANOVA) or Kruskal-Wallis One-Way ANOVA on Ranks (when the variances were considered not equal by Modified Levene Equal-Variance test). Kruskal-Wallis Multiple-Comparison Z-Value Test (Dunn’s Test) was applied when significant differences were found.

Two sets of multivariable logistic regression (MLR) models were performed to investigate the association between periodontitis and obesity using each patient as the unit of analysis – a total of six MLR analyses were performed, three testing the influence of CP on obesity and three testing the influence of obesity on CP. Odds ratio (OR) with a 95% confidence interval were calculated. The first MLR analysis explored the relationship between age, BOP, gender and CP and obesity (the dependent variable was the diagnosis of obesity). The second and third analyses assessed the same independent variables versus the specific diagnosis of obesity. Adipocytokines outcomes’ were not included in these analyses since obese subjects present significant higher serum levels than normal weighted subjects (13).

The fourth MLR model evaluated the influence of age, gender, obesity and serum levels of adipocytokines with CP (the dependent variable was the diagnosis of CP). For the fifth and sixth MLR analyses, the independent variables were the serum levels of adipocytokines; and the dependent variables were the patients with CP + obesity (overall analysis) and patients with CP + obesity presenting sites with PD> 5mm, respectively. For these models, BOP and CAL outcomes were not included in these analyses since subjects with CP present significant BOP and attachment loss than periodontally healthy subjects. The analyses were performed using the NCSS 2007 software package (Number Cruncher Statistical System, NCSS, Kaysville, UT, USA). Differences at *p* < 0.05 were considered statistically significant.

## Results

 Table 1 shows the demographic and periodontal conditions of the four examined groups. There were no significant differences between groups regarding age (*p*>0.05). For CAL, Kruskal-Wallis Multiple-Comparison Z-Value Test confirmed the similarity between NWPH and OPH groups, as well as between NWCP and OCP groups. Also, differences between periodontally healthy and CP groups were confirmed (*P*<0.05). Concerning BOP, obese subjects with CP showed significant more bleeding sites than the other three groups (*p*<0.05). Moreover, subjects from the NWCP and OPH showed similar BOP percentages, as well as OPH group showed more bleeding sites than the NWPH group (*p*<0.05).

**Table 1:**
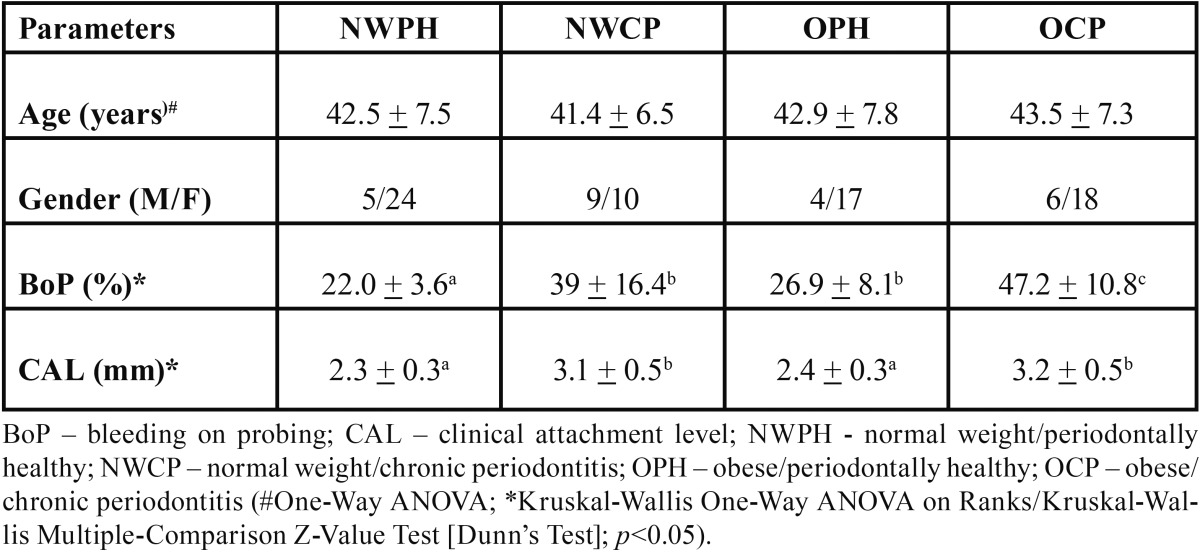
Demographic and periodontal characteristics (mean + standard deviations) of the study population.

With respect to individual adipocytokines outcomes (T Table 2), the OPH group showed similar levels of adiponectin and leptin than the OCP one, but significant higher than NWPH and NWCP (*p*<0.05). However, leptin levels were significant higher for the OCP group when compared to the NWCP group (*p*<0.05). For TNF-α level/]s, there were no statistically significant differences between groups (*p*>0.05).

**Table 2:**
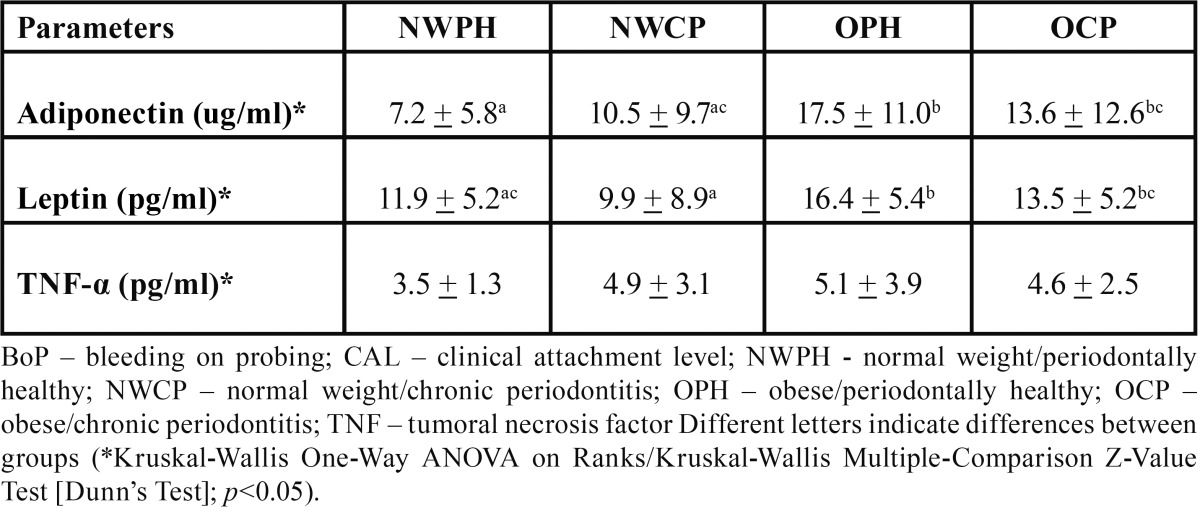
Serum levels of adipocytokines (mean + standard deviations).

The degree of association between obesity and CP was investigated by MLR analyses (Tables  Table 3- Table 6) based on different dependent variables (i.e. diagnosis of obesity, type of obesity [I or II], diagnosis of CP, diagnosis of obesity + CP, and diagnosis of obesity + CP and presence of site exhibiting PD > 5mm). The first and second MLR models found that obese subjects, with CP or not, present significantly more bleeding sites than normal weight individuals with odds ratio of 0.93 (95% confidence interval (CI): -0.88, 0.98 , -0.01; Wald z –value = -2.65; *p* = 0.007) and 0.92 (95% confidence interval (CI): -0.87 , - 0.98; Wald z –value = -2.35; *p* = 0.018), respectively. For the third MLR analyses, a significant association between the type of obesity and the suspected independent variables was not found (*p*>0.05). Regarding the MLR models assessing the influence of obesity on CP, none of them demonstrated statistically significant associations between the suspected independent variables and the tested dependent ones (*p*>0.05).

**Table 3:**
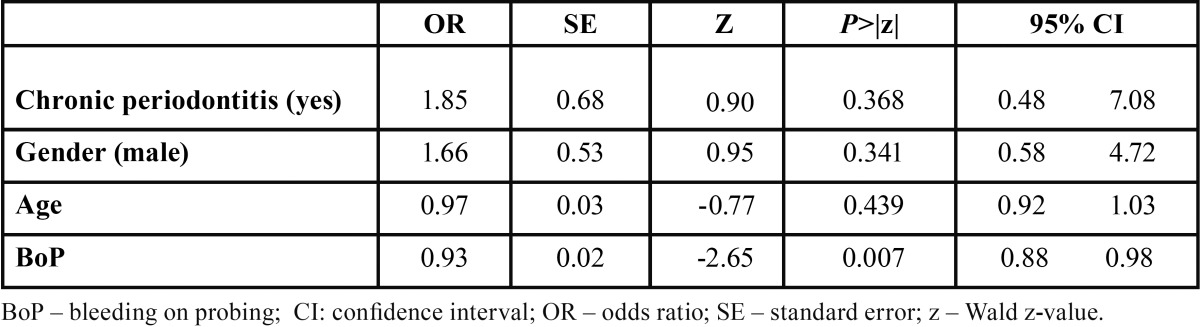
Multivariable logistic regression analysis estimating the interaction effects between periodontal status with a diagnosis of obesity (overall).

**Table 4:**
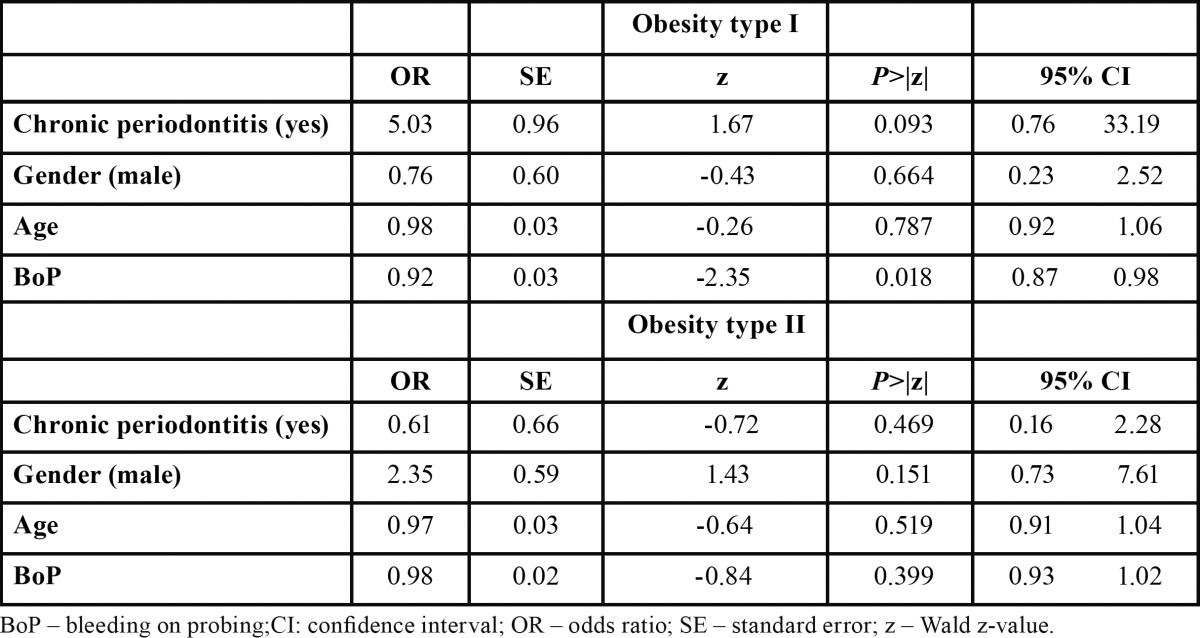
Multivariable logistic regression analysis estimating the interaction effects between periodontal status with a diagnosis of obesity (type I or type II).

**Table 5:**
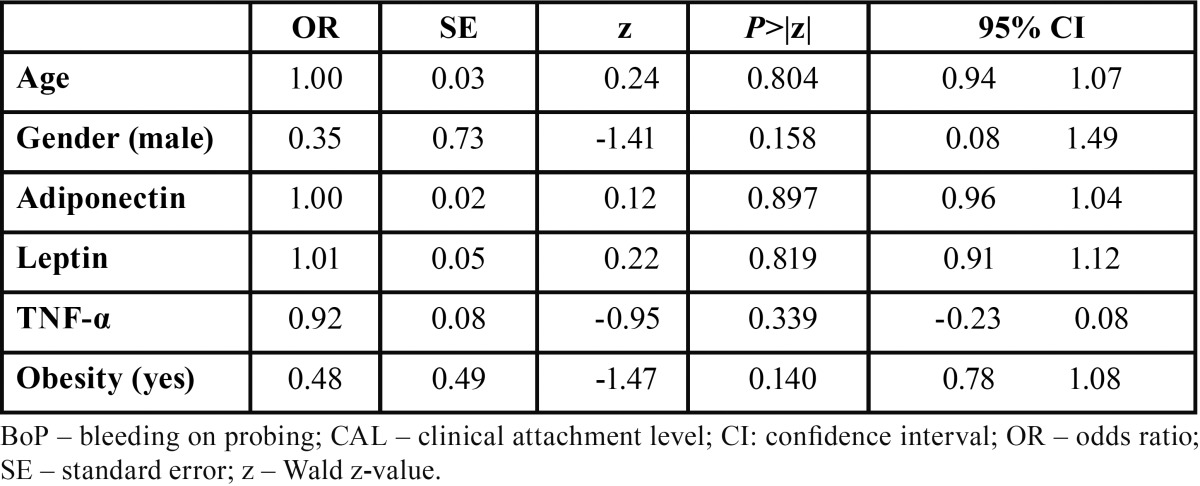
Multivariable logistic regression analysis estimating the interaction effects between age, gender, adipocytokines levels and obesity with a diagnosis of chronic periodontitis.

**Table 6:**
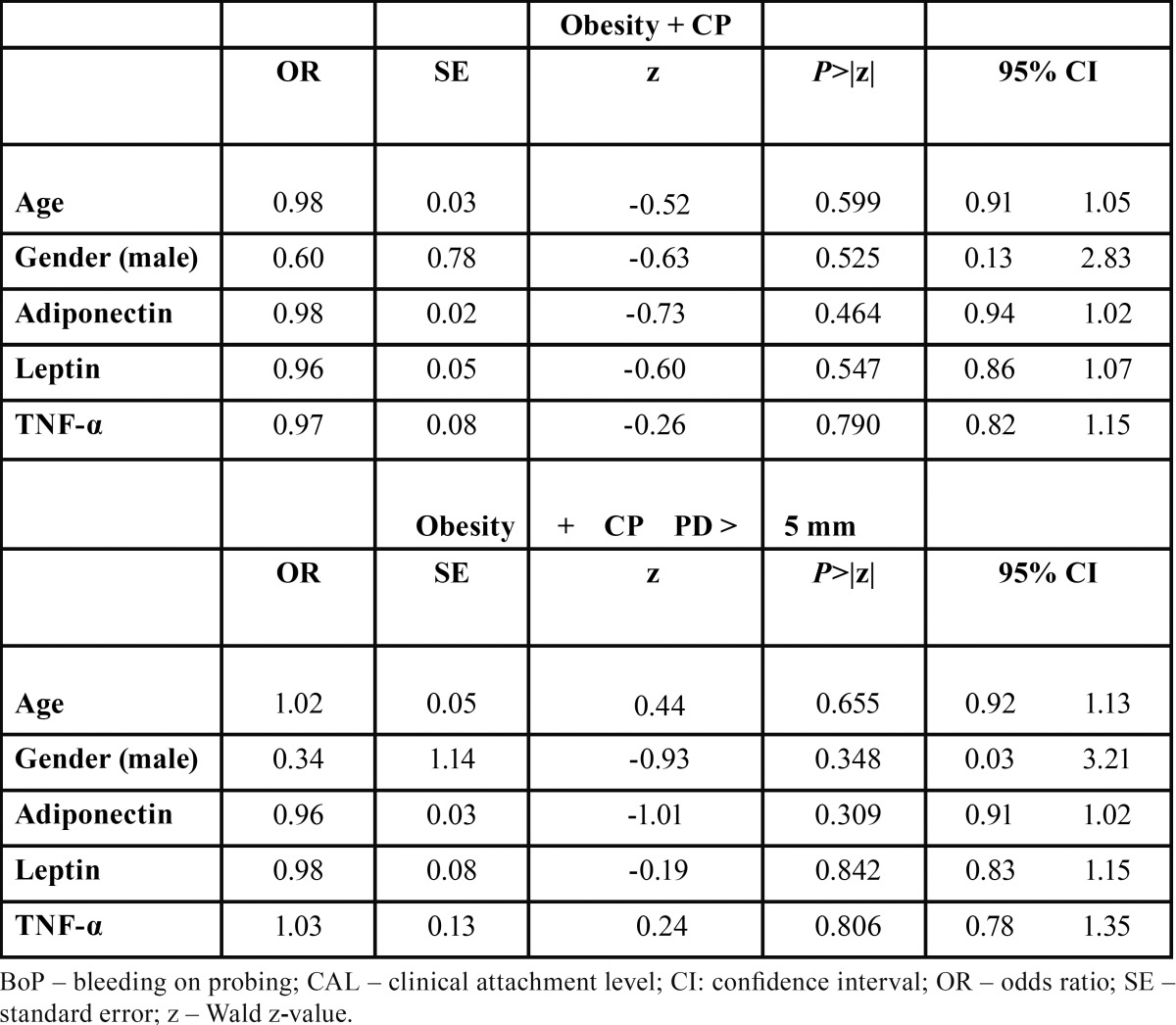
Multivariable logistic regression analysis estimating the interaction effects between age, gender, adipocytokines levels and a diagnosis of obesity + chronic periodontitis.

## Discussion

It may be difficult to accurately predict periodontitis progression if conditions known to influence the host response are also present. Previous studies have investigated the interaction effects with diabetes mellitus and smoking (10,19). Moreover, it has also been considered the potential impact of periodontal disease on systemic health, such as adequate glycaemic control, cardiovascular and renal diseases (19,5-8). Regarding the influence of CP on the levels of adipocytokines, the potential association has been considered an important condition in periodontal research. For instance, a recent systematic review found that subjects with periodontal disease have a 30% greater chance of obesity, as well as higher BMI and CAL loss (18). On the other hand, the same authors did not find prospective observational studies, and thus they considered that it could not be possible to establish causal association (18). The present study failed to sustain these assumptions. Overall, obese subjects with CP presented more bleeding sites than periodontal healthy ones. Moreover, there was a statistically significant equivalency between normal weight subjects with CP and obese ones without CP. CAL was similar between healthy groups and between CP groups, but statistically higher when comparing healthy to CP groups (*p* <0.0001). This finding suggests that obese individuals may be at an increased risk of more severe clinical periodontal inflammation (as measured by BoP) even when a healthy periodontal status is present.

Furugen *et al.* compared the serum levels of different adipocytokines and periodontal status (26) reporting no significant differences in the serum levels of adiponectin and TNF-α between subjects with and without periodontitis. Saito *et al.* did not find asignificant association between periodontitis and adiponectin in middle-aged. Japanese women (27) found significant lower levels of adiponectin in subjects with periodontitis, as well as suggested that this anti-inflammatory marker could be used to periodontal assessment (28) did not find a positive relationship between the serum levels of adiponectin, leptin, resistin, interleukin-6 and TNF-α and CP.

Regarding obesity, to the best of our knowledge, this is the second cross-sectional study designed to assess the relationship between serum levels of adipocytokines and CP in obese and normal weight subjects. Most of the present outcomes are in line with data from the study performed by Zimmerman *et al.* (23). On the other hand, several differences between each individual study`s outcomes deserve some attention: 1) In this study, all groups presented a similar mean age. Zimmmerman *et al.* (23) argued that the levels of adipocytokines could be linked to differences in mean age of their groups instead of being linked to CP, as well as that other factors such as ethnicity and gender, might be related to adipose tissue and adipocytokines` levels; 2) Conversely to the previous publications, all the included patients presented similar ethnicity and food intake (Amerindian-Spanish) what may have assisted in recruiting subjects with similar body fat composition and distribution; 3) Inversely to Zimmerman *et al.* findings (23), the serum levels of adiponectin were similar between obese groups, but higher for OPH when both NW groups were compared (*p* = 0.0009). Moreover, NWCP and OCP groups reported similar adiponectin levels supporting the suggestion that periodontal inflammation seems to regulate the level of this anti-inflammatory adipcytokine as well (23,29). It has been shown that adiponectin regulates inflammation by preventing the stimulation of pro-inflammatory cytokines and allowing the discharge of anti-inflammatory mediators (15). However, previous data suggested that the serum levels of adipinectin seem to be lower in subjects with periodontitis when compared to healthy subjects (26,27); 4) Regarding leptin levels, the lowest leptin levels were seen in normal weight subjects with CP instead of in normal weight subjects without CP, as well as MLR analysis associated obesity to CP (23). It has been shown that leptin leads to an important function in host defense mechanisms because of its role on pro-inflammatory stimuli (30). In fact, both PH groups and CP groups presented similar levels (that is, they were not statistically different), but NWPH subjects showed similar outcomes to OCP individuals, and OPH subjects presented the highest levels (*p* = 0.007). These findings suggest that CP may decrease the serum levels of leptin; and 5) it has been suggested that the higher levels of TNF-α reported to OPH in comparison to NWPH could be partially associated to obesity even without the presence of a periodontal diagnosis. The present outcomes do not support this assumption as no significant differences between groups could be detected (neither the Kruskal-Wallis test nor the MLR models). Earlier data positively linked BMI to TNF-α serum levels (31), but methodological discrepancies, such as the criteria used to assess CP and obesity and the laboratorial tests applied to quantify the serum levels may reflect the differences between samples (23).

In addition, in this cross-sectional study, only a ‘snapshot’ of the potential link between CP and obesity could be analyzed. Also, the diagnosis of obesity was based on BMI alone as proposed by the World Health Organization (2012); however, it has been argued that a diagnosis based on BMI and waist-hip ratio may provide a better characterization of this condition (23). Besides, gingival crevicular fluid was not collected from sites with PD > 5 mm to test for differences between serum levels and locally produced inflammatory cytokines. Due to the limited data available, further studies have to be performed to assess the impact of CP on the serum levels of adipocytokines. Non-significant differences between groups may be associated to type-II statistical error.

## Conclusions

In conclusion and within the limits of this study, it was not possible to establish a positive link between CP and obesity in terms of differences in the levels of adiponectin, leptin and TNF-α between obese and NW subjects with and without periodontitis. However, obese subjects showed significant more bleeding sites than NW ones. In addition, the serum levels of adipocytokines of periodontally healthy obese subjects were similar to normal weight with CP.
